# Implant-Supported PMMA Monolithic Full-Arch Rehabilitation with Surgical Computer-Planned Guide and Immediate Provisional: A Case Report with One Year Follow-Up

**DOI:** 10.1155/2018/9261276

**Published:** 2018-04-01

**Authors:** Vincenzo Luca Zizzari, Gianmarco Tacconelli

**Affiliations:** ^1^Private Practice, Via Benedetto Croce, 85/D, Foggia 71122, Italy; ^2^Department of Medical, Oral and Biotechnological Sciences, University “G. d'Annunzio”, Chieti-Pescara, Italy

## Abstract

The aim of this case report is to describe the surgical and prosthetic procedures to achieve maxillary and mandibular implant-supported PMMA monolithic full-arch rehabilitation (PMFR) with surgical computer-planned guide and immediate provisional. In such cases, the correct planning of dental implants' position, length, and diameter and the prosthetic phases via computer-aided design are very important to achieve good aesthetic and functional long-lasting results.

## 1. Introduction

Edentulism is prevalent worldwide among elderly people, and it is mainly attributed to dental caries and periodontal diseases. However, there is an association between sociodemographic factors, age, gender, low family-income, lifestyle, and tooth loss. In addition, earlier studies have shown edentulism to be a global issue, and it seems to be associated with systemic disorders [1].

When a condition of full-arch edentulism or preedentulism occurs, different rehabilitative options may be chosen: a complete removable denture, an implant-retained removable denture, and an implant-supported fixed prosthesis, either fixed or hybrid [1–4]. The increase in functional demand and social confidence, is leading more and more patients towards the fixed implant-supported options [5].

As regards implant fixtures inserted in edentulous areas, traditional Branemark's protocols recommend 4 or 6 months of submerged and unloaded healing period, respectively, in the mandible and maxilla, after which it is possible to proceed to prosthetic loading, being the process of osseointegration completed [6, 7].

However, in the last few years, the chance to rehabilitate totally edentulous arches through immediately loaded implant-supported prosthesis was found to be a significant opportunity [6–8] due to good success rates and technical simplification introduced by such procedure as widely reported in previous studies [9–12].

Immediate loading is defined as a restoration placed in occlusion with the opposing dentition within 48 h of implant placement [13]. Integration between dental implants and host tissue is strictly dependent upon control of micromovement at the bone/implant interface during the first healing period, and it could be critical in case of immediate implant loading [9, 14, 15]. With full-arch immediate-loading prostheses, the control of micromovement could be achieved only when respecting some conditions: implants should have adequate primary stability at the time of placement, they should be subjected to rigid interimplant splinting, and occlusal forces should be appropriately controlled during the osseointegration period [16, 17].

Computer-aided design/computer-aided manufacturing (CAD/CAM) guided flapless surgery for implant placement using stereolithographic templates is gaining popularity among clinicians and patients [18, 19]. Its advantages are visualization of anatomical hard and soft tissue structures, virtual prototype of definitive prosthesis, accuracy of implant placement, minimally invasive surgical procedures, more predictability, and decreasing time required for definitive rehabilitation. Also, it allows the optimization of implants' position and their parallelism because of the high precision of planning, thus preventing damage to anatomical structures and helping to produce a suitable immediate provisional rehabilitation before surgery [18–20].

The surgical and prosthetic procedures to achieve maxillary and mandibular implant-supported PMMA monolithic full-arch rehabilitation (PMFR) with surgical computer-planned guide and immediate provisional are described below.

## 2. Case Presentation

**Figure 1:**
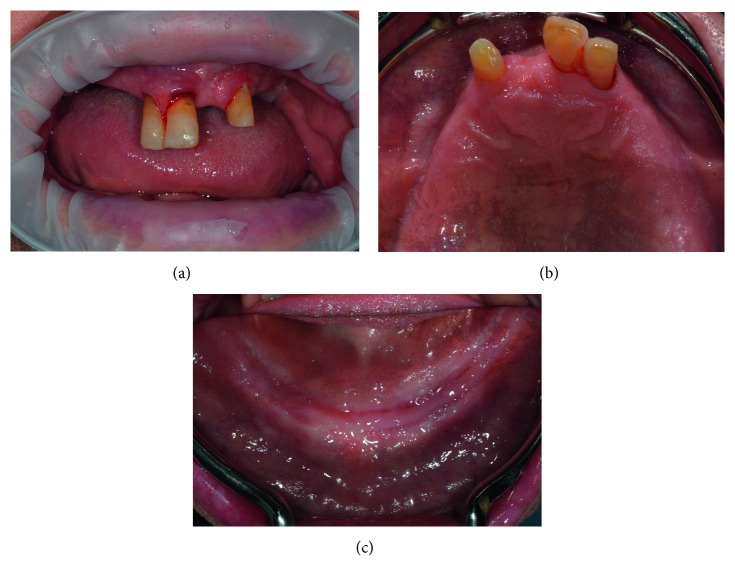
Preliminary clinical evaluation of the patient: (a) maxilla intraoral frontal view; (b) maxilla intraoral occlusal view; (c) mandible intraoral occlusal view.

A 58-year-old man presented few natural teeth in the upper jaw (dental elements 1.2, 1.1, and 2.2) and long-term complete mandibular edentulism (Figure 1). Due to scarce retention of the removable dentures, the patient had not worn them for several years and reported difficulties in eating and speaking, besides aesthetic problems. The patient presented mild hypertension under treatment and antiaggregant therapy and was not a smoker. The primary patient request was a nonremovable rehabilitation. The remaining superior teeth appeared not to be suitable for supporting a fixed rehabilitation, both for their position and for the periodontal attachment loss, and were considered hopeless.

In order to plan an implant-supported oral rehabilitation, a preliminary cone beam computed tomography (CBCT) was prescribed to better evaluate the bone volume of both jaws.

**Figure 2:**
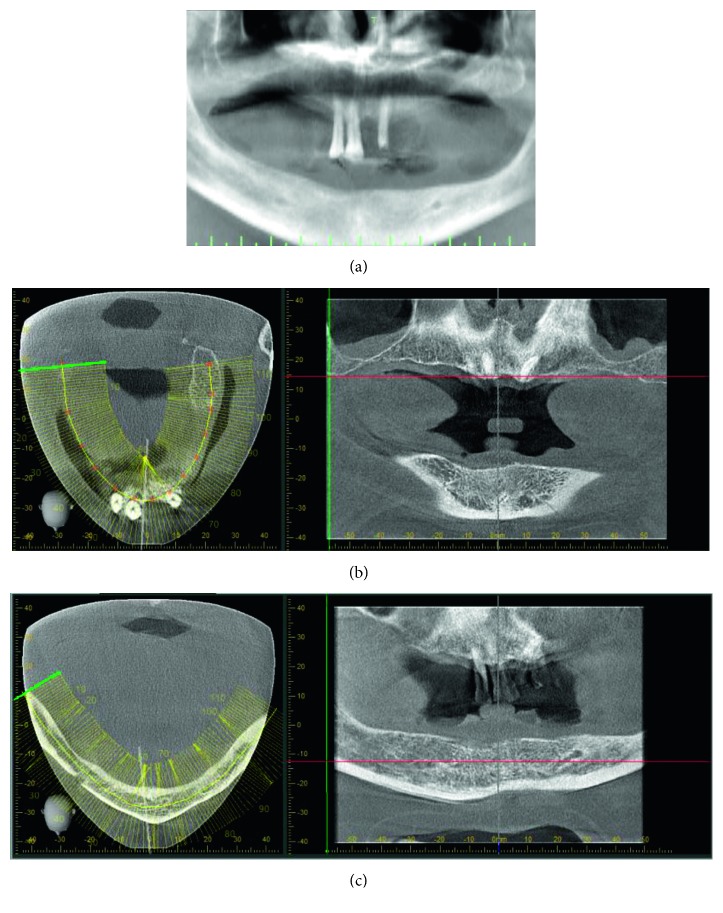
Views of preliminary CBCT evidencing sufficient bone volume in the anterior maxilla and poor bone in the lower jaw except for the interforaminal area: (a) panorex reconstruction; (b) maxilla view; (c) mandible view.

Preliminary radiographic examination through CBCT revealed the presence of sufficient bone volume in the anterior maxilla and poor bone volume in the lower jaw except for the interforaminal area; no periapical radiolucency was discovered in correspondence of the remaining teeth (Figure 2).

The treatment planned consisted in a fixed full-arch rehabilitation of maxilla supported by 5 implants after the extraction of remaining teeth, and a fixed full-arch rehabilitation on 4 implants in the mandible. Both interventions were scheduled to be conducted through a flapless approach after computer-aided planning in order to reduce intraoperative and postoperative morbidity. The strong request of the patient to try everything possible to avoid a provisional mobile restoration led to carefully planning not only implant insertion but also prosthetic rehabilitation. Thus, the patient was informed about the possibility of applying immediate fixed provisional prosthesis if at the time of implant insertions; most of the implants had showed an insertion torque higher than 45 N·cm, as reported by Cannizzaro et al. [21].

The patient was informed about the treatment and a written consensus was obtained according to local legislation.

**Figure 3:**
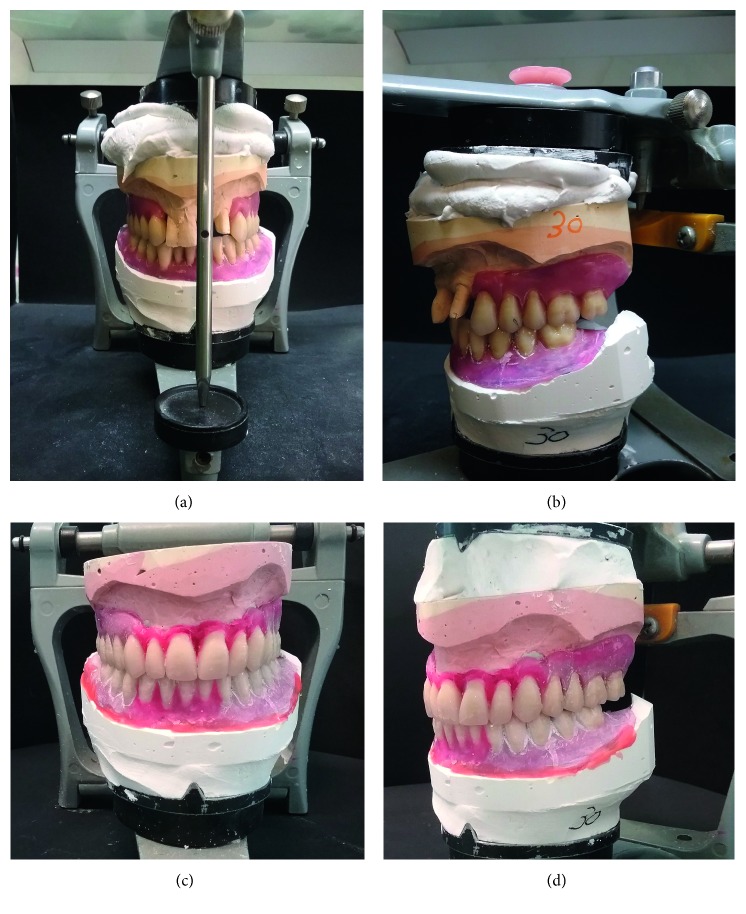
Diagnostic wax-up realized on stone models put in medium-values articulator prior (a, b) and after (c, d) simulating teeth extraction.

Initially, a biphasic impression in vinyl polyether silicone (EXA'lence Putty and EXA'lence Light Body, GC Europe) of both arches was obtained to have initial stone models. After having simulated maxillary teeth extraction on the upper working model, both stone models were put in a medium-values articulator to realize the diagnostic wax-up in order to previsualize the aesthetic result, also considering the extraction of the residual teeth (Figure 3).

As the remaining teeth showed no pathological mobility and their position could not affect the insertion of sufficient number of implants, they could be used for supporting the surgical guide. However, their extraction would be performed on the same day of implant insertion, as they were not necessary for the definitive restoration.

The stone model of the upper jaw was sent to the laboratory, where it was scanned through a model scanner equipped with a computer (7 Series, Dental Wings, Montreal, Canada) to generate STL (STereo Lithography interface format) files. Then, DICOM (Digital Imaging and Communications in Medicine) files derived from preliminary CBCT of the maxilla and STL files from maxilla model scanning were coupled using 3Dyagnosis software (3Diagnosys 4.2, 3DIEMME srl, Italy) so as to be perfectly superimposed, thus obtaining the 3D image that allows the implementation of the intervention planning. Thanks to the presence of the remaining teeth, it was possible to directly couple STL files from the model scan and DICOM files from CBCT using the teeth themselves as reference points, thus avoiding the production of an acrylic guide with radiopaque markers.

**Figure 4:**
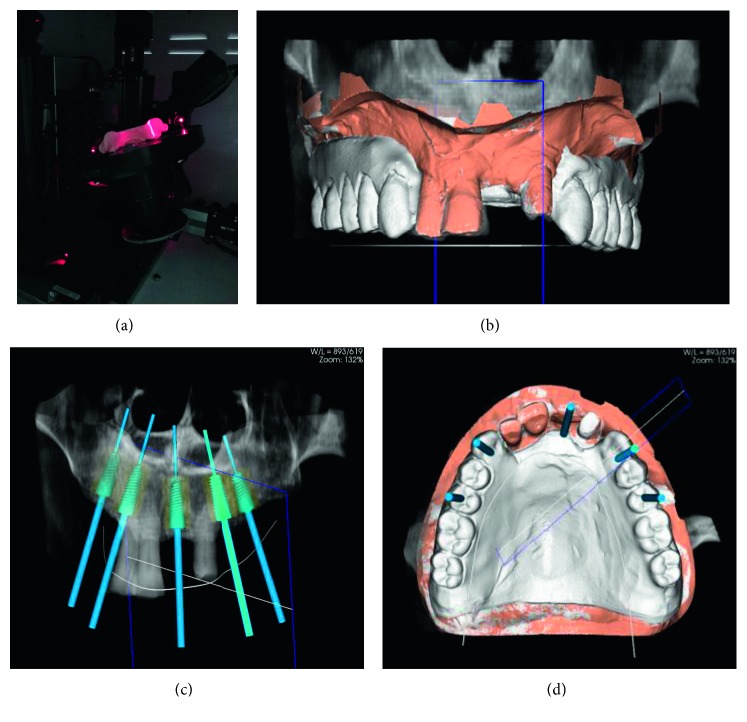
(a) Stone model scanning; (b) coupling DICOM files from CBCT with STL files from model scan; (c, d) computer planning of implant position.

**Figure 5:**
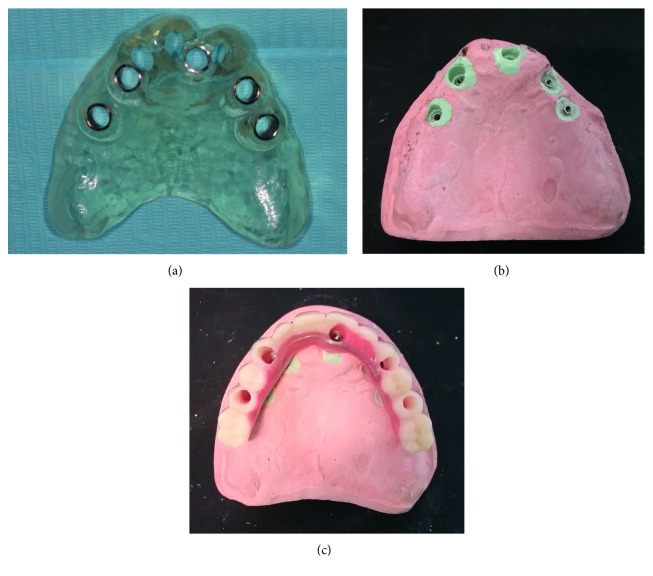
(a) Occlusal view of the teeth-supported maxillary-guided surgical template; (b) maxillary model with analogues inserted in correspondence of planned implant position; (c) maxillary metal-reinforced acrylic provisional prosthesis.

Then, a virtual simulation of the rehabilitation was performed. Once having digitally reproduced the diagnostic wax-up, the position of the implants was planned, considering the bone availability and in a prosthetically driven approach in order to have a favourable emergence of the prosthetic screws (Figure 4). Once the implant position was planned, the project was sent to a CAM center (3DIEMME srl, Italy), and the guided surgical template, being supported by the remaining teeth, was printed by stereolithography in biocompatible material (class I CE). Together with the template, a model with implant analogue holes was provided. In laboratory, the implant analogues were inserted into the model, and a metal-reinforced acrylic provisional prosthesis—1.5 mm diameter steel bar reinforcement—(Acry Pol LL, Ruthinium Group, Badia Polesine, Italy) to be relined on the abutments in the patient's mouth after the implant positioning was produced based on the diagnostic wax-up (Figure 5).

The following preintervention drug therapy was prescribed:

- Antibiotic prophylaxis with amoxicillin 2 g 1 hour before surgery
- Rinse with chlorhexidine 0.20% for 1 minute before surgery

**Figure 6:**
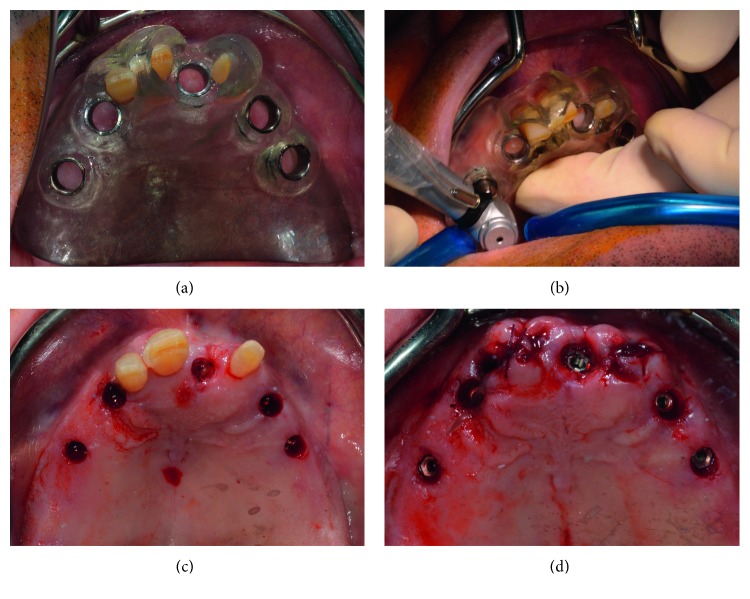
(a) In-mouth try-in of the maxillary teeth-supported surgical template; (b) positioning of the superior dental implants with the aid of the surgical guide; (c) five dental implants positioned in the maxilla before teeth extraction; (d) clinical aspect after teeth extraction.

At the time of surgery, local anesthesia was administered through articaine with epinephrine 1 : 100000, and implant insertion was performed through a flapless approach. After checking the correct seating of the teeth-supported surgical template, soft tissue plugs in correspondence of the sites of implant insertion were removed with the help of a soft tissue punch through the surgical guide. Then, a sequence of calibrated drills with increasing diameters (RealGUIDE surgical kit, 3DIEMME srl) was used to prepare the implant sites under abundant irrigation with refrigerated physiological solution using an implant motor (i-Surge+, Satelec Acteon, France) at a speed of 800 rpm, while implants were inserted mechanically at 25 rpm with no irrigation and under torque measurement control. The implants inserted had external hexagon connection, diameter of 4.2 mm, and length of 11 or 13 mm (MIS Lance Standard Platform; MIS Implant Technologies Ltd., Karmiel, Israel). All the implants showed an insertion torque higher than 45 N·cm, so the remaining teeth could be removed (Figure 6), postextractive alveoli were filled with collagen sponges, and screwed provisional prosthesis was delivered.

**Figure 7:**
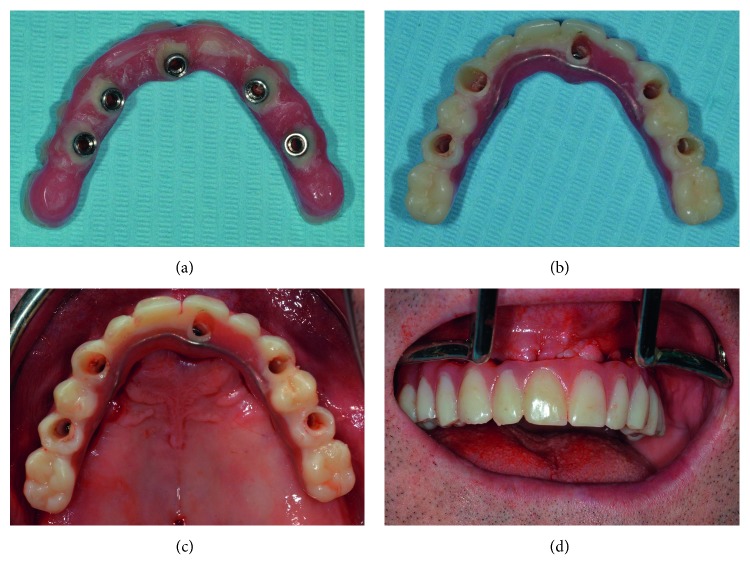
(a, b) View of the provisional upper prosthesis after in-mouth direct abutment fixing; (c) occlusal view of the prosthesis after screwing; (d) frontal view.

In brief, five temporary abutments (Temporary Cylinder Standard Platform, MIS Implant Technologies Ltd.), which height had been previously studied in laboratory in order not to protrude occlusally from the provisional prosthesis, were screwed on the implants, and metal-reinforced resin provisional prosthesis was directly relined and fixed on the abutments with resin (Splintline, Lang Dental Mfg. Co. Inc., Wheeling, IL) and then refined and polished in the dental laboratory and finally placed in the mouth (Figure 7). The provisional prosthesis was screwed on the implants and tightened at 25 N·cm, and then the holes for screws were filled with light-curing resin. Resorbable 4.0 sutures were necessary only at the sites of teeth extraction.

**Figure 8:**
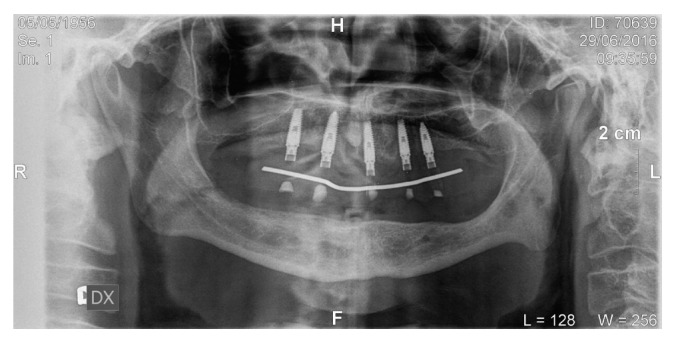
OPG performed after maxillary rehabilitation.

Final orthopantomography (OPG) was performed to control implant insertion and prosthesis fitting (Figure 8). Postintervention drug therapy consisted in

- amoxicillin 1 g twice a day for 5 days,
- ibuprofen 400 mg 2–4 times a day to be taken where necessary,
- rinses with chlorhexidine 0.2% for 1 minute 3 times a day for 2 weeks.

**Figure 9:**
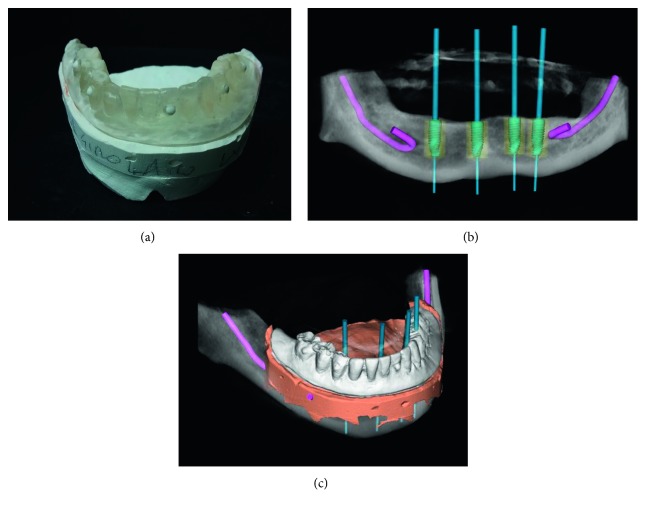
Planning of lower intervention: (a) radiological acrylic template with radiopaque markers; (b, c) virtual planning of implant insertion.

About one month later, impressions of the edentulous mandible and of the maxillary provisional rehabilitation were collected, and a radiological acrylic template with five radiopaque markers reproducing the lower diagnostic wax-up and in occlusion with the maxillary provisional prosthesis was produced. The patient underwent a CBCT of the mandible wearing the radiological template, biting into the established position of centric occlusion. DICOM data from CBCT were processed with the 3Dyagnosis software together with STL files derived from stone model and template scans. The implant insertion was virtually planned as previously described (Figure 9).

**Figure 10:**
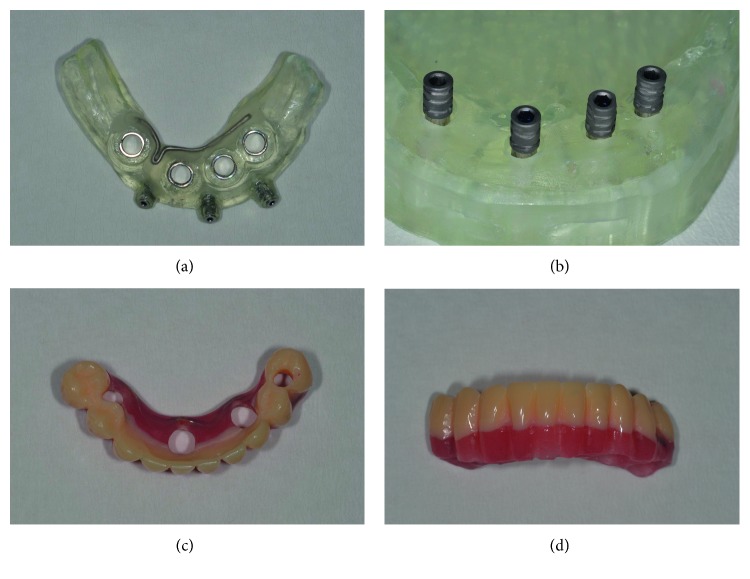
(a) Coronal view of the mandibular guided surgical template; (b) a particular of the mandible model with temporary abutments screwed on the analogues inserted in correspondence of planned implant position; (c) occlusal and (d) frontal views of mandible metal-reinforced acrylic provisional prosthesis.

Moreover, the insertion of three anchor pins for the rigid stabilization of the mucosa-supported guide into its appropriate position during the surgical drilling phase was also planned. Files deriving from the project were processed, and the surgical template, the acrylic model, and the provisional prostheses were produced as described above (Figure 10).

**Figure 11:**
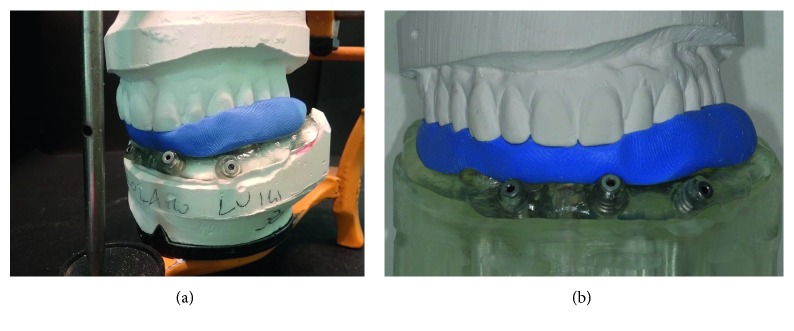
(a) Surgical template seated on the mandible model and put in medium-values articulator for the realization of the silicon index; (b) detail of the three-dimensional relationship between maxilla and mandible models.

In order to provide the correct positioning of the surgical template in the patient's mouth, a silicon index (Occlufast Rock, Zhermack, Badia Polesine, Italy) was realized after seating the surgical guide on the mandible model and putting it in the appropriate three-dimensional relationship to the maxillary model, according to the centric occlusion registered previously (Figure 11).

**Figure 12:**
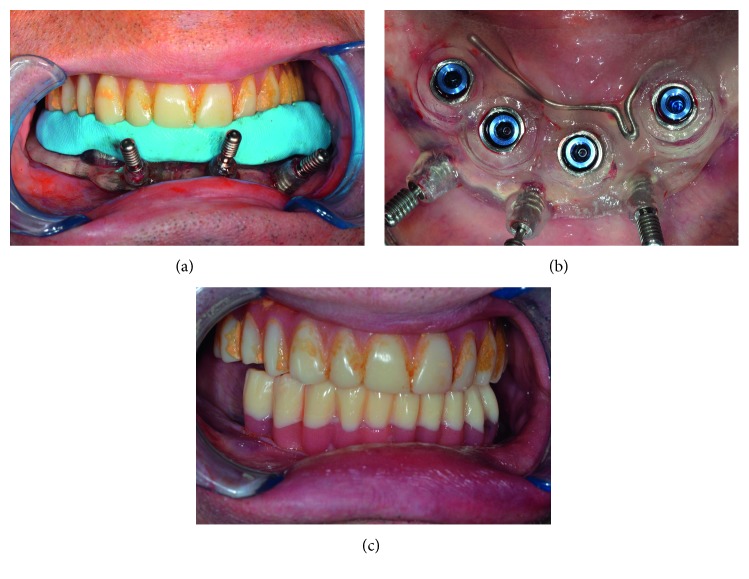
(a) Clinical setting of the template through the silicon index; (b) occlusal view of the inferior implant inserted before removing the surgical template; (c) provisional restorations.

On the day of surgery, after local anesthesia was administered, the correct seating of the surgical template was checked in mouth and, asking the patient to bite the previously obtained silicon index, it was stabilized in its correct intermaxillary relationship through three anchor pins: a 1.2 mm diameter drill was passed through the three vestibular pin holes of the surgical template under irrigation with refrigerated physiological solution using the implant motor at 1000 rpm and the anchor pins inserted. Once the surgical template was stabilized, the silicon index was removed and four external hexagon dental implants, diameter of 4.2 mm and length of 11 or 13 mm (MIS Lance Standard Platform), were inserted following the same procedure as above. No sutures were necessary. As all implants showed an insertion torque higher than 45 N·cm, the patient could receive provisional prosthesis at the same time of surgery, following the same protocol as for the maxilla (Figure 12). Minor occlusal adjustments were performed as required, and the access holes were filled. Preintervention and postintervention drug therapy was administered as described above, and the patient was advised to chew only lightly during the first six to eight weeks after intervention. The patient underwent periodical controls to evaluate mucosal healing and to perform occlusal adjustments to the provisional prosthesis. About four months after the second intervention, biphasic impressions in vinyl polyether silicone (EXA'lence Putty and EXA'lence Light Body) were obtained through individual trays and using the provisional prosthesis as implant transfer, according to the pick-up technique. Moreover, bite registration of the provisional prosthesis was also obtained through a silicon base material (Occlufast Rock).

Impressions were immediately poured to obtain stone models, which were fixed in a medium-values articulator and put in their correct intermaxillary relationship by means of the provisional prosthesis and of the bite registration obtained. Then, articulated stone models were scanned (7Series, Dental Wings), the definitive prostheses virtually designed by the use of a planning software (DWOS, Dental Wings), and related files were transferred to the milling machine.

Definitive PMMA monolithic prosthesis was realized from 98 × 20 mm three-layered PMMA blocks (VIPI BLOCK TRILUX®, VIPI Industria, Pirassununga, SP, Brazil) through a 3D milling machine (DWX-50, Roland DG Mid Europe S.R.L., Acquaviva Picena, Italy), according to the manufacturer's instruction. After milling, the milled pieces were removed from the block by sectioning with a diamond disc, the connection peduncles, and mechanically finished and polished using rubbers and gloss, respectively.

**Figure 13:**
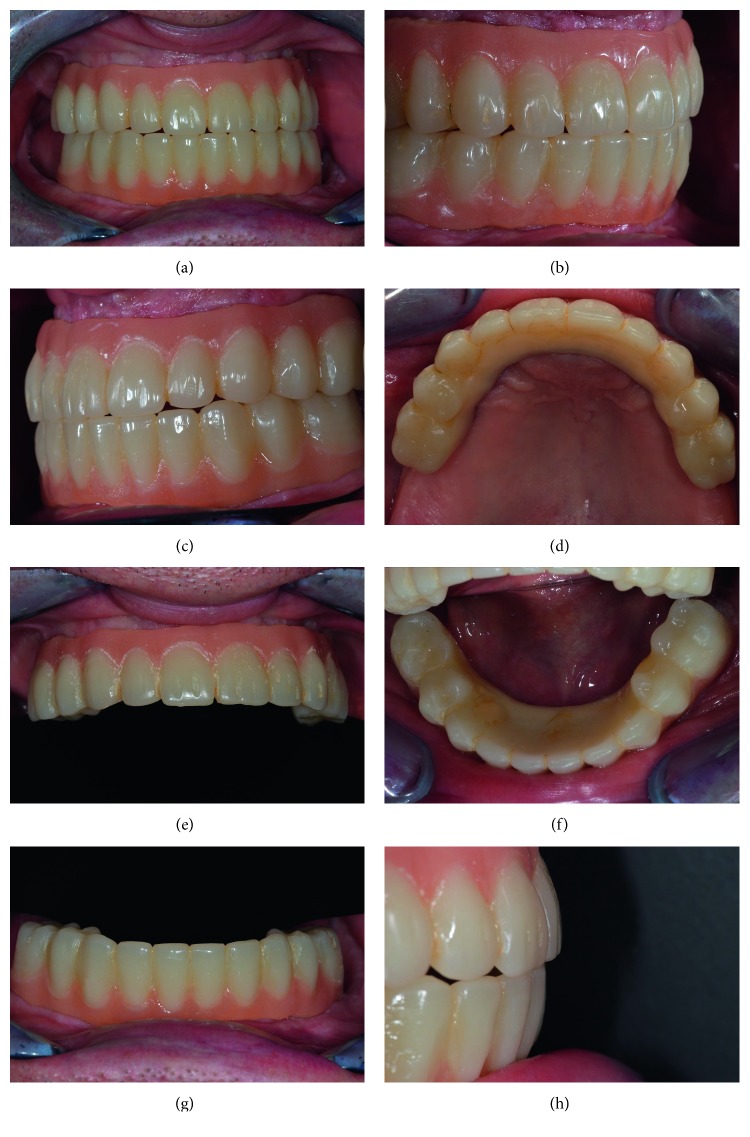
Definitive PMMA monolithic restoration: (a) frontal view; (b) right-side view; (c) left-side view; (d) occlusal view of the maxillary arch; (e) frontal view of the maxillary arch; (f) occlusal view of the mandibular arch; (g) frontal view of the mandibular arch; (h) detail of the frontal bite.

After checking the prosthesis adaptation to the stone models, they were delivered to the patients and connection screws tightened at 25 N·cm; screw accesses were filled with light-curing restoration material (Figure 13).

**Figure 14:**
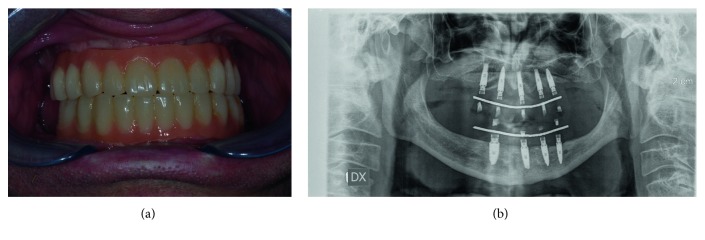
(a) Clinical and (b) radiographical aspect of the definitive restoration after 1 year follow-up.

The patient was instructed about oral hygiene behaviors to follow at home, and periodic controls for in-office hygiene were scheduled every four months. After one year follow-up, the patient was satisfied with the result, with good functional and aesthetical integration of the rehabilitation. Only a few accumulations of bacterial plaque were found on the prosthesis between one control visit and another. Good clinical and radiographic results could be reported, with no sign of soft tissue inflammation and limited bone resorption around the implant necks (Figure 14).

## 3. Discussion

Computer-aided planning of dental implant position is of crucial importance when approaching patients with severe bone atrophy as it allows inserting implants, taking advantage of residual bone and optimizing the prosthetic procedures, as established in previous studies [22–25]. In fact, when the bone volume is really scarce, the only alternative to removable denture could be appealing to more invasive, more time-consuming and sometimes less predictable surgical options, such as guided bone regeneration and maxillary sinus augmentation prior to implant insertion, or the use of zygomatic implants [26–28].

Even if several studies focused on the degree of offset between computer planning and the real position of dental implants [29, 30], other studies confirm the high predictability of more recent 3D planning software and the high level of precision between what is accomplished by the surgeon in respect to what is planned [31].

The use of precise and stable surgical templates also allows clinicians to insert dental implants with a flapless approach [32]. It has already been described how bone tissue exposure during oral surgery causes anoxia damage to the outer bone tissue, resulting in a peri-implant bone resorption [33]. Moreover, a flapless approach is doubtless associated with minor postoperative edema and pain [34], so that it could be considered a preferable approach to treating patients with systemic diseases [35].

The utilization of milling CAD/CAM resins in the fabrication of the definitive prostheses offers many advantages over the use of the traditional acrylic resin. The use of milled PMMA ensures better mechanical properties than using autopolymerizing acrylic resin, due to the lack of polymerization shrinkage [36]. Moreover, the content of residual monomer is significantly reduced, due to the method of polymerization under high pressure that PMMA blocks experience before milling, thus enhancing hardness and wear resistance, decreasing surface worsening and the adhesion of bacterial plaque [37].

A further advantage of using CAD/CAM manufacturing is the data storage and reproducibility of prosthetic device in case of its breaking in short time and with minor discomfort for the patients.

## 4. Conclusion

Even if the described procedure requires a considerable amount of pretreatment planning and the need to attend for CBCT scanning, it could be considered as a valuable and predictable approach to provide full-arch immediate fixed rehabilitation, with many aesthetical and functional advantages especially in cases of severe bone atrophy, where the alternative would be conventional removable denture or more invasive interventions, and in patients with coagulation disorders, who could take advantage from the flapless approach.
